# A peculiar experience– everyday life with chronic sensory disturbances after oxaliplatin treatment for colorectal cancer - a phenomenological study

**DOI:** 10.1080/17482631.2021.1950889

**Published:** 2021-07-23

**Authors:** Birgith Pedersen, Marlene Æ. Jensen, Mette N. Yilmaz, Carsten Dahl Mørch, Casper Feilberg

**Affiliations:** a Department of Oncology, Clinic for Surgery and Cancer Treatment, Aalborg University Hospital, Denmark; Clinical Nursing Research Unit, Aalborg University Hospital, Denmark; Clinical Cancer Research Centre, Aalborg University Hospital, Aalborg, Denmark; b Department of Oncology, Clinic for Surgery and Cancer Treatment, Aalborg University Hospital, Aalborg, Denmark; c Department of Oncology, Clinic for Surgery and Cancer Treatment, Aalborg University Hospital, Denmark; Clinical Cancer Research Centre, Aalborg University Hospital, Aalborg, Denmark; d Department of Health Science and Technology, Center for Neuroplasticity and Pain (CNAP), Integrative Neuroscience Research Group, Aalborg University, Aalborg, Denmark; e Department of Communication and Psychology, Center for Qualitative Studies, Aalborg University, Aalborg, Denmark

**Keywords:** Body perception, colorectal cancer, sensory disturbances, descriptive life-world research, existential phenomenology, Merleau-Ponty

## Abstract

**Purpose:** To deepen the understanding of how survivors’ experience and give meaning to the embodied phenomenon of chronic sensory disturbances in everyday life after oxaliplatin treatment for colorectal cancer.

**Methods:** Data was generated by means of a semi-structured interview guide and drawings with the aim to explore eight survivors’ lifeworld experiences. Data was analyzed through a phenomenological approach.

**Results:** The essential meaning of sensory disturbances emerged in two main themes and four sub-themes. Theme A: ‘A peculiar experience that is difficult to logically understand’ with the subthemes; ‘An ambiguous perception in hands and feet’ and ‘Being alienated from one’s own body’. Theme B: Losing touch with the world’ with the subthemes: ‘A lack of sensory contact with physical surfaces’ and ‘Breakdown of sensitivity in hands hampers fine motor skills and social contact’.

**Conclusion:** Sensory disturbances contributed to an ambiguous and discordant perception of an alienated body that was difficult to describe and affected the ability to act and connect to things and other people. Metaphors and drawings were valuable as means to verbalize and illustrate the changed body perception where the ‘I can’ changed into ‘I cannot’. To support the embodied connection to the world new usage patterns were required.

## Introduction

Overall survival of patients with colorectal cancer (CRC) has improved significantly with current antineoplastic treatments. The state of art treatment includes the chemotherapeutic drug Oxaliplatin that can cause long-term neurological side-effects and chronic sensorimotor disturbances with changes in body perception. However, we still lack evidence about how survivors experience and give meaning to these long-term changes in body perception after this treatment.

## Background

In 2019, 3038 new cases of colon cancer and 1258 new cases of rectal cancer were diagnosed in Denmark (DCCG, Danish Colorectal Cancer Group, 2020). In 2020 it is estimated that 147.950 adults in the USA will be diagnosed with colorectal cancer with a rising incidence in younger people (ASCO, American Society of Clinical Oncology, 2020). A rise is also seen in other developed countries as well as in less developed ones (Costas-Chavarri et al., 2019). Most patients undergo surgery and depending on the stage of the disease, the surgery is followed by treatment with adjuvant chemotherapy using the chemotherapeutic drug oxaliplatin (Costas-Chavarri et al., 2019). Oxaliplatin may influence on the nervous system with sensory, motor and autonomic disturbances and a common side effect is chemotherapy-induced peripheral neurotoxicity (CIPN) with an incidence rate from 38% to 90% (Kerckhove et al., 2017). The symptoms of sensorimotor disturbances may develop rapidly or can appear years after cessation of chemotherapy (Kerckhove et al., 2017). They affect the extremities and include pain, tingling, burning, problems with balance and walking, speaking, swallowing, vision, and tasks that require a high degree of motor control, such as writing and eating. Reduced vibrating sense, touch, proprioception, and progressive loss of deep tendon reflexes have also been identified (Bakitas, 2007; Massey et al., 2014; Stubblefield et al., 2012). Problems including lower limb weakness, impaired proprioception, and functional impairments that compromise the ability to walk or carry items and increased falls, have been less commonly reported (Stubblefield et al., 2012).

How sensorimotor disturbances affect patients in their everyday life after treatment completion was explored in a review that synthesized findings from five studies (Tanay et al., 2017). In this review, two of the studies included survivors after CRC (Bakitas, 2007; Tofthagen, 2010). The functional damage on the nervous system was described as a background noise and a constant reminder of the disease that depended on analgesic efficacy, self-care strategies, increased pain, sleep disturbance, fatigue, and interference with valued activities (Bakitas, 2007). Painful as well as non-painful symptoms that affected the ability to drive, write, collect things, carry out hobbies, household, duties, and exercising, were also reported (Tofthagen, 2010). Three original studies included participants after oxaliplatin treatment for CRC with sensorimotor disturbance from one year up to 16 years (Bennett et al., 2012; Drott et al., 2016; Kanda et al., 2017). Drott et al. (2016) found persistent disturbances after one year and Bennett et al. (2012) found that the symptoms tended to worsen in the first year after treatment completion. These conditions required the survivors to live with sensory disturbances (Bennett et al., 2012; Drott et al., 2016) and highlighted the need for exploring this chronic condition. In addition, the symptoms were perceived worse than expected (Bennett et al., 2012) but were tolerated because the treatment was given with the aim of life prolongation among patients with metastatic CRC (Kanda et al., 2017).

The studies showed how sensorimotor disturbances affected the participants’ everyday lives and complicated their ability to perform everyday activities (Bakitas, 2007). E.g., the survivors had to be watchful when they were doing things (Tofthagen, 2010) as numbness in hands and feet—a non-painful symptom, could lead to unrecognized wounds and thus increased risk of injuries (Bennett et al., 2012; Tofthagen, 2010). The symptoms were difficult to express (Bakitas, 2007; Tofthagen, 2010) and described as fancy, strange and weird (Bakitas, 2007) e.g., *“like the heel and toes are not connecting”* (Bennett et al., 2012, p. 2962). This quotation indicated an affected bodily perception. Additionally, the literature illustrated how the disturbances influenced on the ability to perform everyday life activities.

The ability to perform actions is fundamentally based om embodiment (Carel, 2009), which includes an understanding of the body as a bearer of sensations and a seat of free movement (Grīnfelde, 2018). Suffering from long-term sensorimotor disturbances in everyday living indicate an illness experience which is tied to an embodied perception that may be difficult to understand and express. According to Carel (2012), taking a stance in phenomenology may provide a resource for patients’ ability to reflect on and expand their understanding of their illness. However, none of the studies above applied a phenomenological perspective. To expand knowledge about the embodied experience and to assist healthcare providers to address the specific needs of survivors with chronic sensorimotor disturbances, this study therefore takes a phenomenology approach. From this perspective, the aim of the study is to deepen the understanding of how survivors’ experience and give meaning to the embodied phenomenon of chronic sensory disturbances in everyday life after oxaliplatin treatment for CRC.

### DESIGN AND METHOD

#### Philosophical perspective

The study design is inspired by existential phenomenology (Merleau-Ponty, 2014; Zahavi, 2019). To address the experience of bodily disturbances within a frame of existential phenomenology, the human being is understood as essentially embodied, an inseparable body-subject, termed as *one’s own body* (Carel, 2011; Leder, 1984; Merleau-Ponty, 2004). In addition to be an object that can be weighed and measured, the body is:

‘*the source of subjective feelings, perceptions and sensations; it is the seat of subjectivity, the place where consciousness occurs. As such the body is a subject-object, a unique being that can be experienced both from a first and a third person point of view* (Carel, 2011, p. 37)

Thus, embodiment means that the body becomes the persons point of view (Svenaeus, 2019). Seeing the body as the seat for consciousness, existential phenomenology focuses on what Merleau-Ponty (2014) calls the “I can” rather than an “I think”. Within the body, the world is experienced and understood through perception, and focusing on the “I can”, the bodily capacity to act and move becomes essential.

In health, actions and movements take place unreflectively with a silent and familiar body in an everyday world that are taken for granted—a natural setting for perceptions (Merleau-Ponty, 2004). Perception—the sensory experience of the world, gives access to an immediate embodied experience that appears meaningful and allows human beings to act within our environment. Perception includes the five senses; touch, sight, sound, smell and taste and proprioception—the sixth sense that gives information of body position, movement and acceleration in time and space. It is through these senses we communicate with the world as a familiar place to live in (Merleau-Ponty, 2014).

However, in illness, the ill body parts demand attention and appear in discomfort and the relationship between world and body disrupts as well as habitual routines (Leder, 2021). Thus, the experience of sensory disturbances can never be understood without involving the context of everyday life and life-projects (Carel, 2012; Leder, 1984; Merleau-Ponty, 2004).

### Methodology

In accordance with the philosophical perspective, the analysis is guided by *reflective life-world research*, an open descriptive research method, that aims to describing and elucidating the lifeworld we have access to through our bodies (Dahlberg, Dahlberg & Nyström, 2008). The lifeworld is a natural setting of shared social and cultural meaning, the world we live in, the world we take for granted and typically do not question (Zahavi, 2019). Thus, researching lifeworld is to study everyday life experiences. The manuscript is presented according to the consolidated criteria for reporting qualitative research (Tong, Sainsbury & Craig, 2007).

### Methods

**Figure 1.**
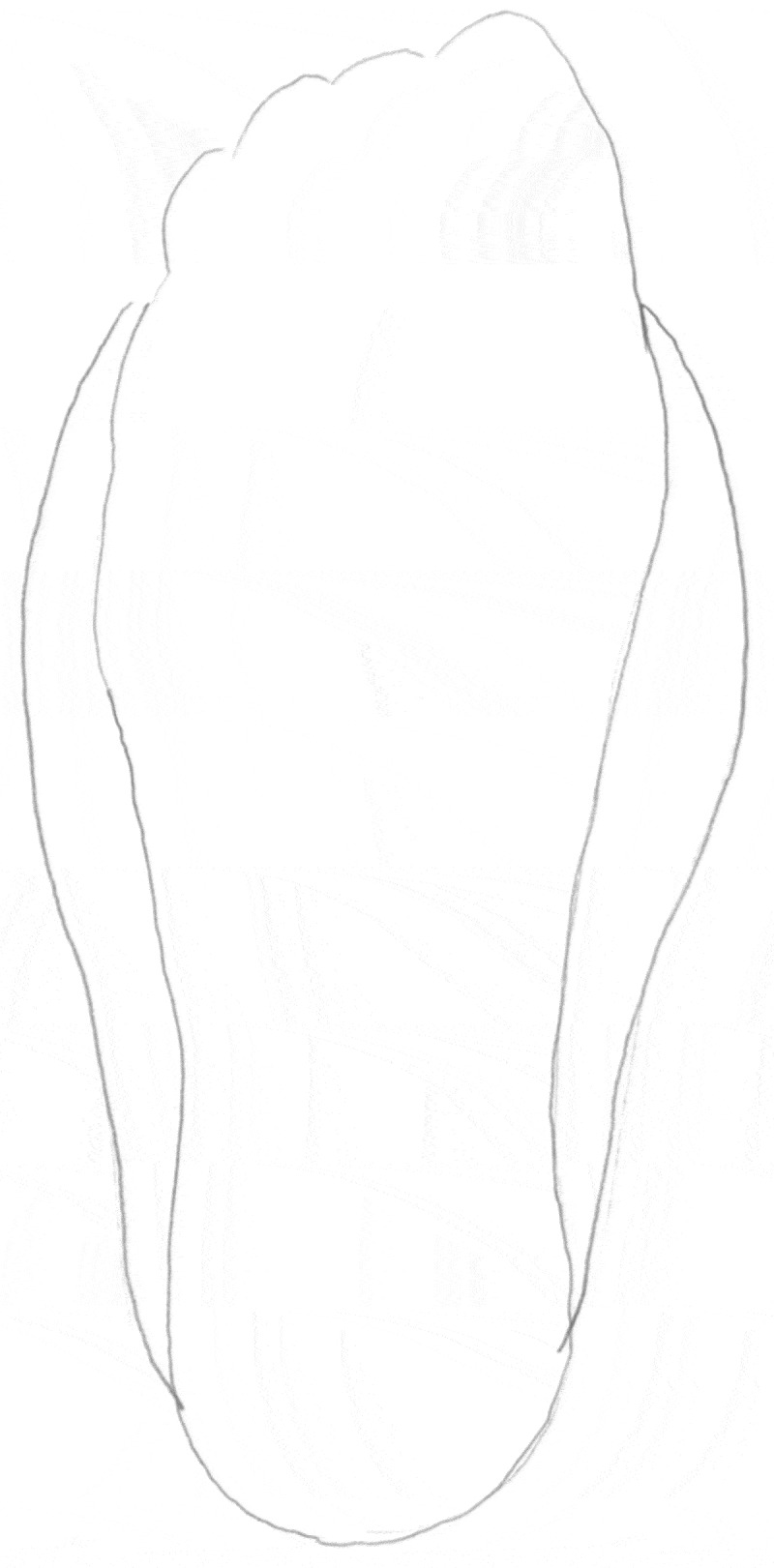
Jack’s drawing of his enlarged foot

**Figure 2.**
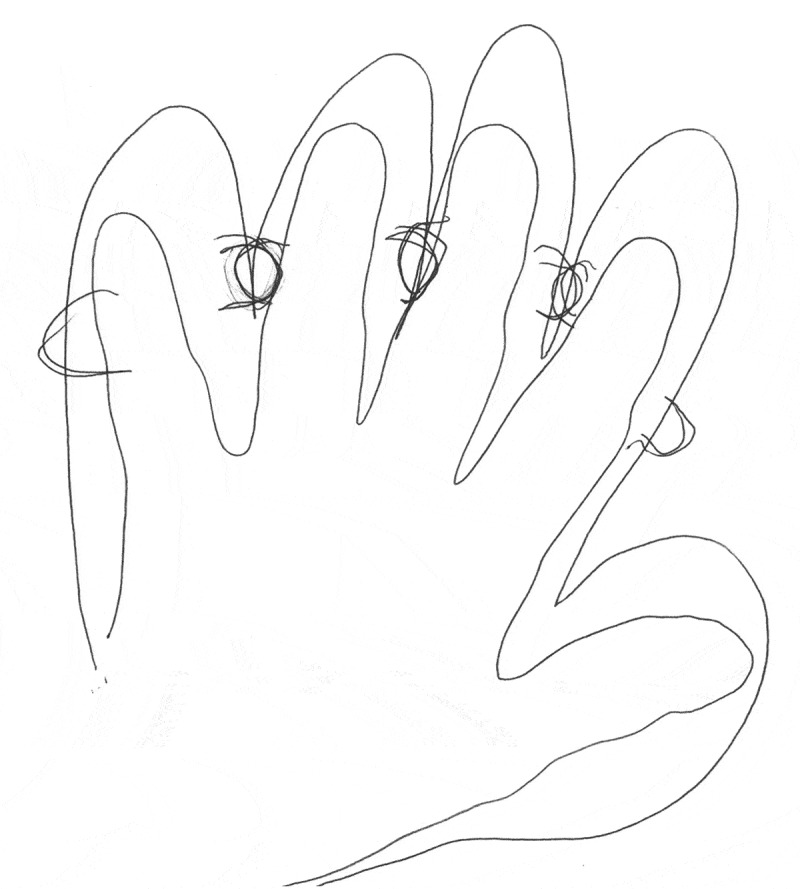
Mary’s drawing of fingers stuck together with knots

**Figure 3.**
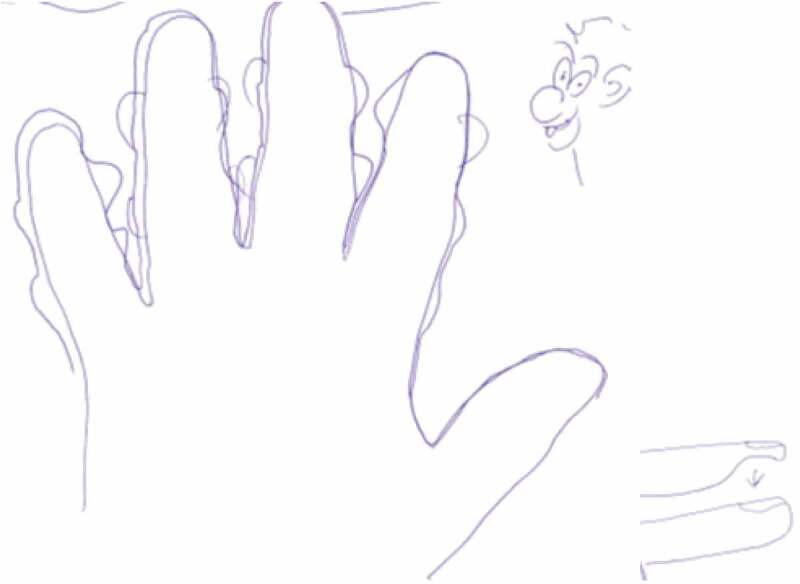
Robert’ s drawing of fingers with knots and half fingertips

The data was generated by a semi-structured interview guide inspired by literature exploring lifeworld experiences (Bakitas, 2007; Dahlberg et al., 2008; Mohrmann, 2017). We posed questions that aimed to explore the influence of sensory disturbances on the participants’ body perception and how the changes influence the ability to plan and accomplish activities that were meaningful in everyday life (Table I). If the participants consented, we additionally requested them to make a drawing of their experiences of changes in hands or feet (Figures 1, 2 and 3). At the wish of participants, five of them were interviewed in their homes and three at the hospital in a convenient room for the purpose. All interviews were conducted and rcorded by the first author alone (nurse with PhD in clinical nursing) between February and May 2019 and lasted from 24 minutes to 64 minutes (mean 38 minutes). The drawing was made after the interview and the dialogue about it was written down immediately afterwards by the interviewer.

**Table I.** Interview guide

1. In your own words, tell me about your experiences of sensory disturbances after completion of chemotherapy and describe some examples of when or how you sense these symptoms. 2. In what way have the symptoms affected your ability to perform everyday actions automatically? 3. When do the symptoms call your attention? Can you give me some example of these experiences? 4. What self-care activities have you used to relieve the symptoms? 5. How do the symptoms influence your perception of your body? 6. How have the symptoms influenced on your relationships with spouses/children, family, friends, colleagues? 7. If possible, could you try to illustrate the changed sensations with a drawing and explain the drawing afterwards? 8. What have we not been talking about that may fulfil your experience of living a life with sensory disturbances after cancer treatment?

### Participants

There is no exact requirement for the number of participants for qualitative and phenomenological studies, but it is expected that data-generating continues until the topic is exhaustedly explored. According to Brinkmann and Kvale (2015), 15 ± 10 is recommended as an appropriate sample. The aim was to gain a rich description of the influence of sensory disturbances on everyday life after cancer treatment for CRC. Therefore, we included eight participants purposefully (Polit & Beck, 2012) among survivors who experienced sensory disturbances after adjuvant treatment with oxaliplatin at least one year after treatment completion. Further inclusion criteria were; age > 18 years, experiencing CIPN in lower or upper limbs, able to verbalize their experiences with CIPN and willing to give informed consent. See Table II for participants characteristics. Participants were excluded if they were unable to read and understand Danish or had pre-existing neuropathy e.g., related to diabetes or neurological diseases. The participants were recruited from the group of survivors after CRC connected to the local unit of the Danish Cancer Society, and from the outpatient unit the Department of Oncology using gatekeepers (the chairman from the local unit of the Danish Cancer Society and clinical nurses from the outpatient unit). None of the approached declined to participate and no relationship between the researchers and participants was established before the study commencement.

**Table II.** Characteristics of participants

Name	Age	Gender	Social status	Working status	Cancer type	Time since treatment cessation(time between interview and treatment)
Dot	76	Female	Living alone	Retired (due to age)	Colon	5 years and 10 months
Jack	67	Male	Living with wife	Retired (due to age)	Rectum	1 year and 3 months
Jean	72	Female	Living alone	Retired (due to age)	Colon	4 years and 6 months
Mary	57	Female	Living alone	Working part time (due to CIPN)	Colon	2 years and 7 months
Karen	63	Female	Living with husband	Working full time	Colon	11 years and 6 months
John	57	Male	Living with wife	Early retirement (due to CIPN)	Rectum	7 years
Robert	69	Male	Living with wife	Retired (due to age)	Colon	3 years and 9 months
Carl	62	Male	Living with wife	Early retirement (due to CIPN)	Rectum	6 years and 3 months

### Ethical considerations

The study complies with the ethical guidelines for research of The Nordic Nurses Federation (Nordic Nurses Federation, 2003) and Helsinki Declaration (WMA, The World Medical Association, 2018). Participants’ were informed about the project orally and in writing and signed informed consent before the interview. Participation was voluntary, and the participants were informed about their right to withdraw from the study at any time without consequences. In Denmark qualitative research does not require ethical approval, but the project was notified to the Danish Data Protection Agency (ID 2018 150). The data is stored in a safe place only accessible to the researchers and confidentiality and anonymity are insured by coding data and exchanging names with pseudonyms.

### Analysis

To claim scientific rigour for the analysis based on existential phenomenology, some conditions need to be met (Dahlberg et al., 2008; Omery, 1983; Todres, 2005). In accordance with our philosophical perspective, the phenomenological analysis aims to laying out participants’ perception of sensory disturbances as closely as possible to their embodied experience of the phenomenon. This involves a phenomenological method or attitude (Merleau-Ponty, 2004; Zahavi, 2019). Stepping into a phenomenological attitude requires an awareness of our natural attitude, the taken for granted (Zahavi, 2019). Thus, we strove to become aware of our preconceived ideas, our habits of thoughts, our prejudices, and theoretical assumptions in data gathering as well as in the analysis and interpretation of data by putting them into play during discussions in the research group. As a complete reduction of our pre-assumptions is impossible (Merleau-Ponty, 2014), they were bridled to ensure they only supported a better understanding of the participants’ experiences (Dahlberg & Dahlberg, 2019; Dahlberg et al., 2008).

Initially, we transcribed the interviews verbatim. Next, four interviews were read and discussed in the research group. The group consisted of two nurses experienced in oncology and with, respectively a master’s degree in clinical nursing and a PhD degree in clinical nursing, a medical doctor and team leader for the colorectal staff, a psychologist and a biophysicist, both associate professors employed at an university. Staying open for what appeared significant in the participants’ descriptions (Dahlberg et al., 2008; Sadala & Adorno Rde, 2002; Zahavi, 2019), the discussion challenged each member of the group’s pre-assumptions and theoretical concepts and contributed to an immediate impression of the essential meaning of the participants’ sensory disturbances.

To structure the data, essential meaning units from the interview text were identified and coded to form clusters of meanings (Table III). The meaning units were clustered across all eight interviews by the two nurses after which all authors contributed to form patterns in main themes and sub-themes until consensus was reached (Table IV). The findings were laid out in a descriptive analysis in the findings section. Finally, the essential meaning, described in main—and subthemes were further interpreted and discussed with theory and other research findings in the discussions section (Dahlberg & Dahlberg, 2019).

**Table III.** Structuring data—the coding process

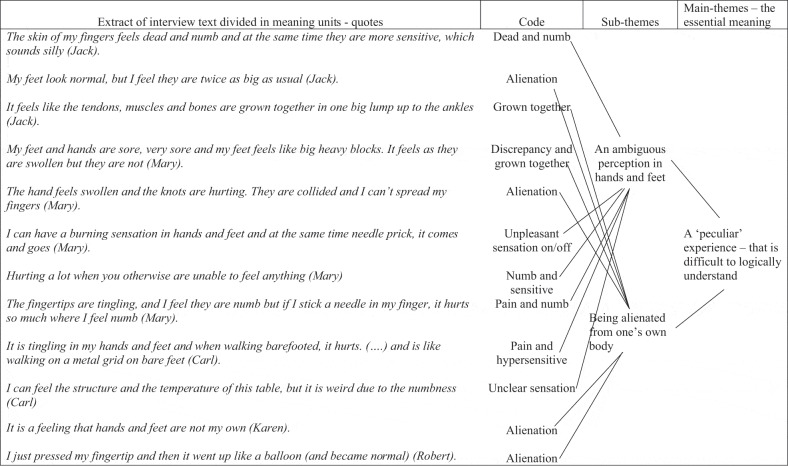

**Table IV.** Extract of clustered meaning units with essential themes of meanings

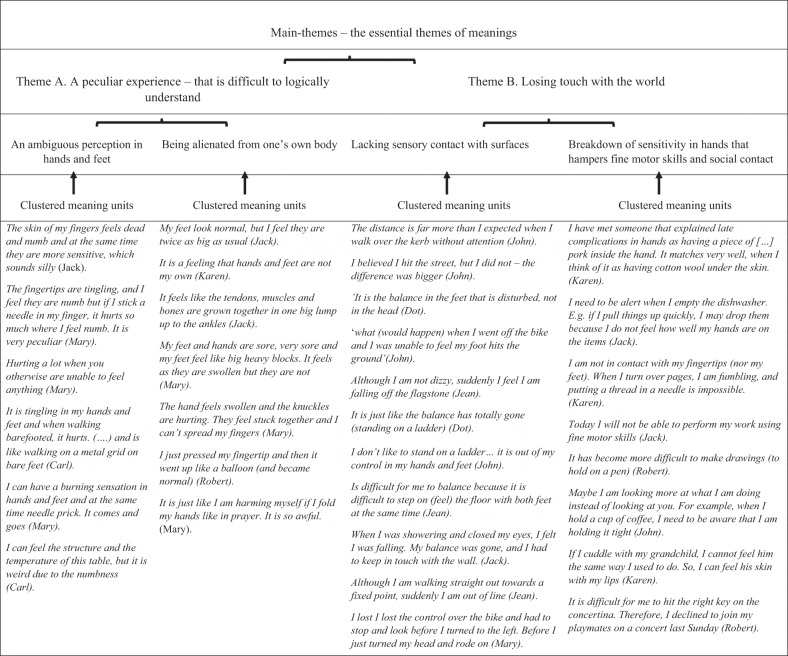

## Findings

**Figure 4.**
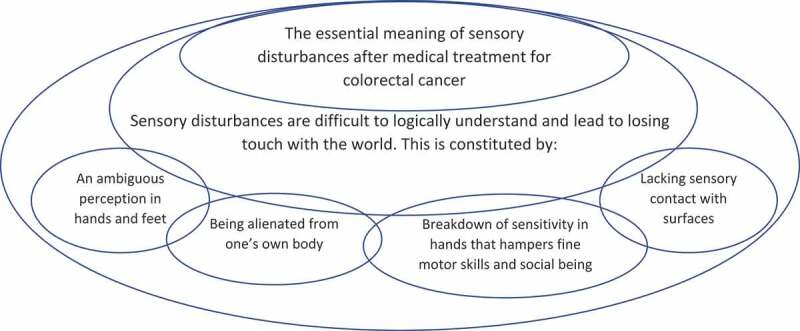
The essential meaning of sensory disturbances

The analysis resulted in two main themes both constituted by two sub-themes in a structure of meanings that is illustrated in Figure 4. The first theme revealed how sensory disturbances appeared as “A peculiar experience—that is difficult to logically understand” because it differed from former everyday experiences and appeared as a discrepancy between appearance and sensation. The theme was elaborated in the subthemes, “An ambiguous perception in hands and feet” and “Being alienated from one’s own body”. The second theme, “Losing touch with the world” illustrated a breakdown with how life used to be and the challenge of returning to a new life adapting the bodily changes due to lack of sensory contact with physical surfaces, things and the skin of self and other people either in hands or feet. The theme was elaborated in sub-themes, “A lack of sensory contact with physical surfaces” and “Breakdown of sensitivity in hands hampers fine motor skills and social contact”.

### Main theme A. A peculiar experience—that is difficult to logically understand

The participants expressed the view that the changed body perception was difficult to describe and articulate to others. They were confronted with an ambiguous and discordant sensation especially in the hands and feet, e.g., these parts of the body were perceived as numb and at the same time hyper-sensitive. In addition, the perception of the sizes and the shapes of hands and feet differed from how they appeared visually.

#### Sub-theme A1. An ambiguous perception in hands and feet

Due to being difficult to understand, the sensory disturbances were described as silly and peculiar, as they included an odd feeling in hands and feet with different perceptions at the same time. To provide the interviewer with an expression of the disturbances, the participants compared their sensory disturbances to the experience of frozen finger and toes that were warming up and with numbness and tingling. One participant described the odd feeling like this, *it is peculiar. The skin of my fingers feels dead and numb and at the same time they are more sensitive, which sounds silly* (Jack). The same experience was elaborated by other participants. John and Mary described an experience of numbness of the outer skin. At the same time, they experienced intense pain as if the skin was penetrated by a piece of broken glass or a needle. The ambiguous feeling was further described by Mary in this way:

*The fingertips are tingling, and I feel they are numb. It is funny, if I stick a needle in my finger, it hurts so much where I feel numb. It is very peculiar, I think (laugh). Hurting a lot when you otherwise are unable to feel anything. This is very peculiar (laughs)* (Mary).

Painful sensation occurred in the numb hands and feet not only when the skin was penetrated. The experience of numbness and pain at the same time also appeared when walking on rugged surfaces or just walking barefoot. Mary sensed her numb feet and hands as burning with pinpricks inside—but the feeling in feet appeared when she walked on rough surfaces. Carl expressed his experience in this way. *‘it is tingling in my hands and feet and when walking barefooted, it hurts. It is not like walking on broken glass but more like walking on a metal grid on bare feet* (Carl).

Apparently, the increased sensations and peculiar feelings became noticeable when performing bodily actions and e.g., strain was put on the feet. However, the participants were unable to understand their experiences from a logical and common-sense point of view in their endeavour to describe different aspects of their experiences and making sense of what appeared contradictory.

Thus, the participants tried to articulate an experience of blurred and a painful perception that is much more undifferentiated and ambiguous than they normally can express and give meaning to. To share their peculiar experience with others, the participants used pictures or metaphors to describe their sensory disturbances.

#### Subtheme A2. being alienated from one’s own body

In addition to the ambiguous perception in hands and feet, the participants described a discrepancy between how feet and hands appear visually and how they are perceived. Although feet and hands were looking normal, the feet felt enlarged with the skin being too tight and bones growing together, hands with painful knots and feeling unable to spread the fingers or close the hand.

Jack expressed his experience of the discrepancy between reality and his feeling as follows:

*My feet look normal, but somehow, I feel they are twice as big as usual. It feels like the tendons, muscles and bones are grown together in one big lump up to the ankles* (Jack).

Jack also made a drawing of his enlarged feet that felt twice as big as they were. However, he felt that this drawing was not adequate to illustrate his peculiar perception as he was unable to draw a three-dimensional picture and show how his ankles were affected (Figure 1). Mary felt her feet as being like heavy, sore blocks. Additionally, she gave words to the feeling of inconsistency between appearance and sensation regarding her odd, enlarged hand by saying:

*My feet and hands are sore, very sore and my feet feel like big blocks. Heavy blocks and sore. It feels as if they are swollen but they are not. The hand feels swollen and the knots are hurting. They are stuck together, and I can’t spread my fingers – it hurts, and it is just like I am harming myself if I fold my hands. It is so awful* (Mary).

A drawing helped Mary to express her feeling (Figure 2). This picture showed the normal-looking hand surrounded by an outline that illustrated the clumsy, and lumpy perception of the same hand. Thus, it seemed that the discrepancy induced an alienation, which was also illustrated by Karen; *it is a feeling that hands and feet are not my own* (Karen). However, reflecting on her own drawing Mary suddenly understood her perception of her sensory disturbances and mysterious enlarged hands on a deeper level. Now it occurred to her why she had trouble spreading her fingers because, as shown on the drawing, she perceived them as having grown together. In addition, she understood her pain when she saw the big knots in her finger joints that were stuck together and said, *now I better understand.*

Robert also contributed a drawing (Figure 3). Besides big hands with knots, he experienced that his fingertips were half size, and to make them feel normal, he explained; *I just pressed my fingertip and then it went up like a balloon (and became normal)*(Robert).

The experience of a discrepancy between appearance and perception with respect to their hands and feet that made them feel bigger and lumpy, and fingertips as half-size seemed to push to a new experience of alienation with respect to parts of their body that is introduced after the cancer treatment. In the worst case, this peculiar experience made Karen question if her hands and feet were her own at all, which signalled not only alienation from a well-known body but a divided body where hands and feet as strange objects not belonging to the unified body-subject.

### Main theme B. Losing touch with the world

In main theme B, we describe how the sensory disturbances change the way bodily actions take place unreflectively and disturb the habits and the trust in one’s own bodily capacity for action. Thus, the changes require an increase in attention on performing everyday activities in order to compensate for the sensory disturbances.

#### Subtheme B1. A lack of sensory contact with physical surfaces

The participants described problems with balance, especially when biking, standing on a ladder and taking a shower with closed eyes. However, upon closer inquiry, the feeling of missing balance originated from a lack of contact with the physical surfaces. Something had affected their ability to sense their own body and feel the surfaces. John said:

*When I walk over the kerb, without attention to the difference in level, suddenly, the distance is far more than I expected. Something that comes naturally for other people (but not for me because) I believed I hit the street, but I did not – the difference was bigger. Damn it, it hurts* (John).

The difficulty with feeling the surface extensively influenced John’s everyday life. Several years went by before he had the courage to go biking again after his treatment. His concerns about biking were related to a lack of sensory contact with the surfaces, because “*what (would happen) when I went off the bike and I was unable to feel when my foot hits the ground”* (John). However, to compensate for this, he learned to recognize the tightness of the upper leather of his shoes when they hit the ground.

For other participants, the lack of contact with surfaces was described as losing the sense of balance. However, the lack of balance was not associated with dizziness but described as a feeling of losing the balance in hands and/or feet, *’It is the balance in the feet that is disturbed, not in the head* (Dot). The interpretation of losing one’s balance seemed to be associated with not being able to feel the surfaces with the feet like before cancer treatment. The changed perception in feet is further described by Joan who loved to take a walk at the beach and compensated for what she interpreted as losing one’s balance with walking sticks. She said:

*When I take a walk, I always use sticks (… .) however, it is very unpleasant. Although I am not dizzy, suddenly I feel I am falling off the flagstone* (Jean).

According to Jean, she did not feel a general dizziness, but when her contact with the ground through the skin of her feet betrayed her, she suddenly experienced a sense of falling. The sticks enabled her to walk at the beach, because they help her rebalance and sense the ground through the hands via the sticks. However, not only the contact with the ground through the feet was affected according to participants’ descriptions, the contact between hands/fingers and the touch of things and other people was also affected.

#### Sub-theme B2. Breakdown of sensitivity in hands hampers fine motor skills and social contact

In addition to disturbed sensation in their feet, participants described a changed sensitivity in their hands. The numbness, tingling and dead feeling in addition to having cotton wool under the skin of the hands signalled losing touch with world. Karen added the following metaphor to describe the affected sensitivity in her hands:

*I have met someone that explained late complications in hands as having a piece of […] pork inside the hand. It matches very well, when I think of it as having cotton wool under the skin*. (Karen)

Following Karen’s explanation, a new sort of barrier had arisen between the sensory hands and the surfaces. This new sensory restrictedness caused problems in the interaction with things and surfaces and the ability to touch as usual when using fine motor skills like separating pieces of paper, picking up small pieces, holding a glass, cups and items or playing musical instruments. Thus, due to the barriers experienced in the hands, the participants experienced a breakdown of their sensitivity described by Jack:

*I need to be alert when I empty the dishwasher. E.g., if I pull things up quickly, I may drop them because I do not feel how well my hands are on the items*. (Jack)

The sensory disturbances required the participants to be more observant when they performed everyday activities and kept a heightened level of attention, which signalled a potential distrust in normal habitual body actions like holding a cup or glass. To increase their intentional awareness, they used vision to compensate for the lack of sensations in the hands. Robert, who loved to play the concertina was unable to feel the keys as usual and had to replace it with a wind instrument that enabled him to see the keys. John further explained:

*Maybe I am looking more on what I am doing instead of looking at you. For example, when I hold a cup of coffee, I need to be aware that I am holding it tight (John)*

In addition to the increased attention on how to drink a cup of coffee, the quote from John indicated that the awareness on performing tasks with changed sensations in the hands affects his ability to be in eye contact with people sitting in front of him and potentially affected the way he connected to other people. For Karen, the meaning of the changed perception in hands was expressed as; *I am not in contact with my fingertips (nor my feet). When I turn over pages, I am fumbling, and threading a needle is impossible* (Karen). The lack of contact with surfaces extensively influenced her contact with her grandchild. So not only her contact with inanimate surfaces and activities that require fine motor skills but also her ability to create bonds to her family through her hands was restricted. Losing tactile sensation that used to be a matter of course, she remembered how it used to be when feeling a little child’s skin and said:

*(… .) if I cuddle with my grandchild, I cannot feel him the same way I used to do. So, I can feel his skin with my lips, but … I remember how it felt to touch a little child, but the feeling is no longer available in my hands (Karen)*

In the quote above, it appeared that Karen found another way to create a physical contact with her grandchild that compensated for the breakdown in sensations she experienced in her hands. However, it also signalled a longing for the former body perception—where action could take place without the requirement for compensating.

To summarize, the participants describe a lack of sensory contact with physical surfaces, things and the skin of themselves and other people. The missing sensory contact was disturbing, and the participants compensated by increasing their attention during the activity, or by using tools (sticks) and their lips to develop the contact, that is missing in the situation. For the participants, losing sense experience that used to be a matter of course, became a picture of how life used to be and the challenge to return to a new life by adapting to bodily changes. Although it seemed that the participants had adapted to their sensory disturbances, they expressed lack of trust in their own bodily actions.

## Discussion

### The peculiar experience

The aim of the study was to deepen the understanding of how survivors’ experience and give meaning to the phenomenon of chronic sensorimotor disturbances in everyday life after oxaliplatin treatment for colorectal cancer. Our findings revealed that the sensory disturbances appeared as a peculiar experience that was difficult to describe and understand as the participants felt ambiguous sensations in their hands and feet, e.g., as being numb and at the same time hypersensitive. These findings correspond with the findings of Tofthagen (2010), that reported a combination of painful and non-painful sensory and motor disturbances that affected everyday living in several ways within three years after treatment completion.

The contradictory sensation of increased pain and numbness at the same time may be explained from a neurophysiological point of view. The oxaliplatin-induced toxicity may affect the nerve fibres and jeopardize the gate-control mechanism which inhibits pain information at the spinal level (Melzack & Wall, 1965). In addition, it seems the small sensory nerve fibres being affected has decreased the number of nerve fibres innervating the skin (Krøigård et al., 2014). Paradoxically, the nerve fibre loss coincides with increased activity in small sensory nerve fibres that may cause central sensitization and thus increased pain perception and numbness at the same time (Woolf, 2011). Even though the sensory disturbances were understandable from a neurophysiological point of view, the contradictory sensations in hands and feet did not correspond with the participants’ previous understanding or common sense of either being numb or hypersensitive. Although it is through sensing meanings are recognized (Leder, 1984), it seemed that the participants were unable to make sense of what appeared unintelligible and incoherent. The parts were not joined to a meaningful wholeness (Dahlberg & Dahlberg, 2019), which corresponds with Bennett et al.’s (2012) description of no connection between heel and toes.

Furthermore, the discordance made it difficult to express the bodily sensation to other people, that is consistent with the findings of Bakitas (2007) and Tofthagen (2010). The vocabulary the participants used in our study was “peculiar” and “odd”, which adds to the way the participants described their sensations as fancy, strange and weird in Bakitas’ (2007) study. However, in the attempt to incorporate the apparently meaningless sensation into meaningfulness (Svenaeus, 2011), the embodied experiences were described with examples from everyday life and by use of metaphors. Revealing these embodied experiences in the participants’ stories, they unfolded a meaning—a picture of an embodied existence with sensory disturbances as a condition for everyday living. The taken for granted was changed. The body was no longer absent and silent (Leder, 1990; Merleau-Ponty, 2004) but now situated in an everyday life with persistent ambiguous and discordant perceptions in hands and feet that indicated an ongoing illness experience.

Being familiar with one’s own body affects the way the unified body-subject is anchored in the world and how the world appears meaningful (H. Dahlberg, 2019; Svenaeus, 2011). However, in illness our lifeworld as a well-known home territory takes an un-homelike character (Svenaeus, 2011). Although the sensory disturbances had become an everyday embodied condition, the discrepancy between the appearance of hands and feet and the experience of enlarged feet and hands with painful joints and knots induced a perception that some parts of their bodies did not belong to them. The division of the unified body-subject where hands and feet became “them” opposed to the rest of the body, indicates an alienation from one’s own body, that according to Svenaeus (2011) contributes to an experience of an unfamiliar and homeless body—“*a being me and not yet me*” (Svenaeus, 2011, 2019, p. 463). Thus, not being acquainted with *one’s own body* influenced the fundamental understanding of a unified, inseparable body-subject, which instead turned into a thing—an unfamiliar being and a disintegration of body and self (Leder & Krucoff, 2008). However, making drawings and explaining them for the interviewer seemed to push to an understanding of the sensation of body parts that looked normal but were experienced as unfamiliar and alienated. The statement, “*now I better understand …* ” indicates that this kind of actions may help the unified body-subject to grasp a new understanding that may contribute to turning the still strange body into a familiar body. Thus, combining different ways to explore sensory disturbances may contribute to a deeper understanding of sensory disturbances not only for survivors but for healthcare professionals, as well.

### Losing touch with the world

Experiencing lack of sensory contact with surfaces, balance problems and being hampered in their fine motor skills, the participants bodies no longer remained in the background or disappeared as a silent familiar and functioning body. Although the participants did not suffer from observable body-disfigurements, the body became present in its disability and “dys-appeared” as an injured body figure in the foreground (Dahlberg, 2019; Slatman, 2014). Dys-appearance is a mode of being that refers to an explicit body awareness, where the former silent body appears disabled and dysfunctional (Leder, 1990). According to Merleau-Ponty, in illness, disease or disabilities, *“it is as though our bodies comprises […] two distinct layers, that of the habitual body and that of the body at this moment* (Merleau-Ponty, 2004, p. 84). The habitual body acts as a reference for the actual body (Merleau-Ponty, 2014). In our study, the participants were restricted in carrying out everyday actions due to limitations in their actual body. Their “I can” was disrupted because their illness interfered with the balance between ability and inability, which according to Leder (1990, 2021) has the tendency to change “I can” into “I cannot”, meaning “I no longer can, as I once could”.

Although the interviews illustrated, how the participants were limited in their everyday living, they also showed how the participants coped with their altered bodies. Suffering from chronic disturbances made them unable to restore and alter to the habitual body that they remembered from the past. Instead they used a strategy of transforming, which is a way of developing new usage patterns that enable the emergence of a new “I can” (Leder, 2021). When they were unable to accomplish motor control due to lacking the sense of different levels of the street or strained not to drop a cup or a glass, they sharpened their attention of their body position and ability to move—their sixths sense, which is connected to the sense of touch (Leder & Krucoff, 2008; Radcliffe, 2008). However, they had lost their former way of being in touch with the world as the sensory disturbances affected their sensory contact with physical surfaces and induced a breakdown in sensitivity due to the reciprocal character of touching. The reciprocal character of touch means that touching, is to be touched back whatever it is about touching another person or touching surfaces (Leder & Krucoff, 2008). Affected by the reciprocity of touching and being touched, the participants kept attention on how to use their fine motor skills, where to put their feet not to fall off the flagstone, sensed with other parts of the body when getting off the bike or used walking sticks to compensate for the disturbed sensations. Thus, using tools and keeping increased attention tended to become a new normal to overcome the sensory disturbances. However, losing touch with the world, not only the touching agent but the touched changed and the everyday unreflective “being—in—the world” seemed to break down (Leder & Krucoff, 2008; Merleau-Ponty, 2014). In addition, with bodily disturbances, an increased attention on performing tasks may influence the ability to direct attention outwards to other people (H. Dahlberg, 2019). In our study, we found examples of how the relationship to other people was changed or weakened because the connection between body and world no longer was taken for granted. The unity of the body-subject was broken as the silent dis-appearing body that acted unconsciously with embodied skills, dys-appeared with sensory disturbances. Drinking a cup of coffee that before took place unreflectively, now required an attention that prevented the participant in keeping eye contact as usual. Furthermore, loss of sensory contact with the skin of the little grandchild had the potential to affect the intimate relationship and connection between grandmother and child. The influence of sensory disturbances on social contacts seems to be a potential risk as touch is a metaphor for social connection—to be in touch not only physical but emotional and psychosocial (Leder & Krucoff, 2008). In that sense, the affected sense of touch and proprioception affected the whole embodied connection to the world.

### Strengths and limitations

In discussing the trustworthiness of the findings based on reflective life-world research, remaining in a scientific phenomenological attitude as required by Norlyk and Harder (2010) supports the rigour and trustworthiness of a study. We emphasized discussions in the research group to confer impressions and ideas and prevent premature closures during the analysis as well as inputs from different professions and competencies challenged the group members pre- assumptions. Thus, the credibility and dependability of the findings were strengthened (Dahlberg, 2006; Dahlberg et al., 2008; Norlyk & Harder, 2010). Using a semi-structured interview guide with explorative follow-up questions, the participants narrated their experiences from their natural attitude providing rich description with variations on the topic that allowed the subsequent presentation of the essential meaning structures. However, the applicability or transferability of the findings to other contexts must be judged by the reader (Norlyk & Harder, 2010). Thus, the findings have the potential to add to the understanding of the influence of sensory disturbances related to other diagnosis or treatments and independent of where in the healthcare system the provision of care takes place. In this sense, the findings move beyond the specific context of survivors after colorectal treatment and healthcare professionals working in oncological departments.

### Conclusion

Taking a philosophical stance in existential phenomenology combined with a life world methodology in analysing and discussing survivors’ experiences of sensory disturbances after CRC treatment revealed a first-person perspective that contributed to new knowledge of how these disturbances influenced everyday life. The meaning of the sensory disturbances appeared ambiguous and discordant associated to experiencing an alienated body that was difficult to describe. In order to express this alienation of the unified body-subject, metaphors and drawings were valuable as means to verbalize and illustrate lack of coherence between bodily perception and visual impression on periphery parts of the body (hands and feet). Lacking the feeling of contact with surfaces in hands and feet influenced the participants’ ability to act and connect to things and other people and was a breakdown in everyday unreflective being. Their “I can” changed into “I cannot”. To cope with the changes and minimize the illness experience of sensory disturbances, they sought to develop new usage patterns to support the embodied connection to the world.

### Implications

A phenomenological approach can disclose how illness, pain, discomfort etc. influence on human beings “being in the world” (Carel, 2012; Dahlberg, 2019; Slatman, 2014; Svenaeus, 2011). Investigating changes in perception from neurophysiological perspective or by questionnaires is relevant but not enough to cover the survivors’ experiences that are full of meaning and significance. The present study has contributed to revealing how sensory disturbances affect a person’s embodied being in the world and shows that they have far-reaching consequences on survivorship after treatment for CRC. However, this study is only a step on the way to giving voice to these serious implications. The findings require healthcare professionals to pay attention to a perspective on embodiment and the lived experience of patients in the endeavour to facilitate patient-centred care. However, we still lack more knowledge of how the alienated body may influence on the survivor’s self-perception and relationships with other people.
